# NAFPD exacerbation by hyperlipidemia combined with hyperuricemia: a pilot rat experiment in lipidomics

**DOI:** 10.3389/fnut.2024.1437373

**Published:** 2025-01-07

**Authors:** Jingyun Li, Yongjian Chen, Shilin Li, Guorong Lyu, Furong Yan, Jiajing Guo, Jing Cheng, Yun Chen, Jiaojiao Lin, Yating Zeng

**Affiliations:** ^1^Department of Ultrasound, Second Affiliated Hospital of Fujian Medical University, Quanzhou, Fujian, China; ^2^Department of Medical Imaging, Quanzhou Medical College, Quanzhou, Fujian, China; ^3^Department of Molecular Diagnostics Research Center, Second Affiliated Hospital of Fujian Medical University, Quanzhou, Fujian, China; ^4^Department of Pathology, The 910th Hospital of the People's Liberation Army, Quanzhou, Fujian, China; ^5^Department of Animal Experimental Center, Quanzhou Medical College, Quanzhou, Fujian, China; ^6^Department of Internal Medicine, Quanzhou Medical College, Quanzhou, Fujian, China

**Keywords:** hyperuricemia, lipidomics, non-alcoholic fatty pancreas disease, non-alcoholic fatty liver disease, glycerophospholipids

## Abstract

**Background:**

Hyperuricemia and non-alcoholic fatty pancreas disease (NAFPD) are prevalent metabolic diseases, but the relationship between them remains underexplored.

**Methods:**

Eighteen Sprague–Dawley rats were randomly assigned to three groups: normal (CON), high-fat (PO), and high-fat high-uric acid (PH). After 12 weeks, serum uric acid (SUA) and triacylglycerol levels were measured. Pathological changes in the pancreas were assessed using hematoxylin–eosin (HE) staining. Serum samples were analyzed using lipidomics technology, and multivariate statistical analysis was employed to identify differences in lipid metabolism.

**Results:**

SUA levels in the PO group were not significantly different from those in the CON group (*p* > 0.05). However, from the 4th week onward, SUA levels in the PH group were significantly higher than those in both the PO and CON groups (*p* < 0.05). HE staining revealed that most rats in the CON group exhibited normal pancreatic islet and acinar cell morphology. The pathological NAFPD score in the PH group was higher than that in the PO group. Lipidomics analysis identified 34 potential serum biomarkers in the CON and PO groups, 38 in the CON and PH groups, and 32 in the PH and PO groups. These metabolites primarily included sphingolipids, cholesterol esters, fatty acids, triacylglycerols, phosphatidylcholines, lysophosphatidylcholine, phosphatidylethanolamine, and lysophosphatidylethanolamine.

**Conclusion:**

Hyperlipidemia combined with hyperuricemia might exacerbates NAFPD. Glycerophospholipids may serve as key biomarkers in this process, potentially linked to a chronic inflammatory response mediated by glycerophospholipids.

## 1 Introduction

The concept of “excessive pancreatic fat” was first introduced by Ogilvie in 1933. Non-alcoholic fatty pancreas disease (NAFPD) is primarily characterized by pancreatic fat infiltration, also known as pancreatic steatosis. Despite its recognition, the pathophysiology of NAFPD remains incompletely understood, and no unified standard diagnostic criteria or consensus has been established. The prevalence of NAFPD varies significantly, ranging from 12.9 to 16% in China (1), 33% in New Zealand (2), and up to 61.4% in Korea (3). NAFPD is strongly associated with diabetes, atherosclerosis, acute and chronic pancreatitis, and pancreatic cancer (4). Given its high morbidity and link to various diseases, understanding the underlying mechanisms of NAFPD is crucial.

Hyperuricemia (HUA), a metabolic disorder defined by elevated serum uric acid (SUA) levels, has become increasingly prevalent due to changes in dietary habits. HUA is the primary cause of gout but may also act as an independent risk factor for metabolic diseases such as hypertension, non-alcoholic fatty liver disease (NAFLD), and diabetes (5, 6). NAFPD and NAFLD often occur together, suggesting shared pathophysiological mechanisms (7, 8). Moreover, HUA is common in patients with NAFLD (9), and uric acid (UA) levels correlate with the progression and severity of NAFLD (10–12). For instance, Choi et al. (13) demonstrated that UA stimulation promotes lipid accumulation in HepG2 cells and mouse liver cells, while studies on animal models have shown that UA-lowering treatments such as allopurinol and benzbromarone improve hepatic lipid deposition (14). However, research on the direct effect of HUA on NAFPD remains limited.

Since the mid-1990s, metabolomics has emerged as a pivotal field in biomedical research, complementing genomics, transcriptomics, and proteomics in elucidating biological processes (15). Metabolomics enables the extraction, separation, analysis, and identification of small-molecule endogenous metabolites—such as amino acids, lipids, and sugars—in biological samples such as blood, urine, cells, and tissues. These metabolites, often bioactive at low concentrations, provide critical insights into the body’s metabolic characteristics under various physiological and pathological conditions. They are also valuable as potential biomarkers for disease diagnosis and progression. Lipidomics, a specialized branch of metabolomics, focuses on the dynamic metabolic profiles of endogenous lipids in response to external stimuli (16, 17). Recent advances in lipidomics have deepened our understanding of diseases such as cardiovascular conditions, cancer, diabetes, neurodegenerative disorders, and liver diseases, while facilitating the discovery of novel biomarkers (18, 19). Lipid metabolites are increasingly recognized for their clinical potential, especially in the context of personalized healthcare and precision medicine (20). Loomba et al. demonstrated that polyunsaturated fatty acids could serve as non-invasive biomarkers for diagnosing non-alcoholic steatohepatitis using the liquid chromatography–mass spectrometry (LC–MS) platform (21). In a rat model, Zeng et al. (22) identified the creatine-to-betaine ratio as a potential diagnostic marker for early liver cancer. Additionally, Meikle et al. highlighted the strong association between plasma phospholipids, sphingolipids, and other lipid metabolites with cardiovascular event risk, suggesting lipid biomarkers for monitoring interventions such as statin therapy to assess cardiovascular disease risk (23). However, no definitive serum markers for NAFPD have been established. Although pathological diagnosis remains the gold standard for NAFPD, pancreatic biopsy is challenging due to the pancreas’s deep location and the procedure’s high risk. Consequently, non-invasive serum markers with simple testing procedures have significant practical value.

This study aimed to preliminarily explore the relationship between HUA and NAFPD through animal experiments. A classic NAFPD model was established using a high-fat diet and a high-fat, high-uric acid model with a high-fat, high-yeast diet combined with potassium oxonate. Using these models, SUA and triacylglycerol (TAG) levels were measured, pancreatic changes were evaluated using light microscopy after HE staining, and serum lipid metabolites were analyzed using lipidomics technology based on high-performance liquid chromatography-triple quadrupole mass spectrometry (HPLC-QqQ-MS). This study intends to identify potential biomarkers and provide a theoretical foundation for further NAFPD research.

## 2 Materials and methods

### 2.1 Materials

All solvents used were of LC–MS grade. Acetonitrile, isopropanol, methanol, and water for mass spectrometry were purchased from Merck (United States), and methyl tert-butyl ether was obtained from Sigma (Germany). The internal standard mix was subscribed from the Internal Standards Kit for Lipidyzer™ Platform (SCIEX, MA, United States).

### 2.2 Establishment of hyperuricemic rat model

Male Sprague–Dawley rats (250–300 g) were sourced from Shanghai Slake Experimental Animal Co. Ltd. and housed at the Animal Experimental Center of Quanzhou Medical College (China). The experiment was approved by the Ethics Committee of the Second Affiliated Hospital of Fujian Medical University (No. 2019–125).

Animal models were established using a modified version of the Mazzali method (24, 25). Eighteen Sprague–Dawley rats were randomly assigned to three groups: a control group (CON), a high-fat group (PO), and a high-fat, high-uric acid group (PH). The groups were fed diets tailored to each model: the CON group received a standard diet, the PO group was given a high-fat diet (45% fat by calories), and the PH group was provided with a high-fat, high-uric acid diet containing 3% potassium oxonate and 20% yeast powder (45% fat by calories). All rats were housed in a controlled environment (temperature: 18–20°C, humidity: 50–60%) for 12 weeks. Blood samples (approximately 1 mL) were collected from the tail at weeks 4 and 8 for analysis of serum uric acid and triglyceride levels. At week 12, the rats were anesthetized with 2% sodium pentobarbital (40 mg/kg) through intraperitoneal injection. The amount of sodium pentobarbital could be increased appropriately, making sure that the rats did not respond to pain. Once unresponsive to pain, the rats underwent laparotomy, and approximately 3 mL of blood was drawn from the heart. The pancreas was then quickly excised. Rats did not survive after cardiac blood sampling.

### 2.3 Measuring of serum biochemical indicators

After standing at room temperature for 30 min, whole-blood samples were centrifuged twice at 4,000 rpm for 5 min, and the supernatant was collected. Serum uric acid and triglyceride levels were measured using commercial assay kits from Nanjing Jiancheng Biological Engineering Co. Ltd.

### 2.4 Pathological manifestations in the pancreas

Pancreatic tissues were fixed in 4% paraformaldehyde, and pathological changes were evaluated under a light microscope following hematoxylin and eosin (HE) staining. The study extended the work of van Geenen et al. (26), assessing four indicators: pancreatic islet cell vacuoles, pancreatic acinar vacuoles, interstitial fat infiltration, and interstitial inflammatory cell infiltration. Each index was rated as normal (1 point), mild (2 points), or moderate (3 points) based on the extent of fat infiltration. Scores for each indicator were summed to yield a final pathological score.

### 2.5 Serum lipidomics analysis

#### 2.5.1 Lipid extraction

For lipidomics analysis, serum samples were prepared using the isopropanol method as described by Sarafian et al. (27). The process involved the following steps: (i) precooling isopropyl alcohol and other organic reagents at −20°C; (ii) thawing 20 μL of serum at 80°C and transferring it to an EP tube; (iii) adding 400 μL of isopropyl alcohol and thoroughly mixing the samples for 3–5 min with a vortex mixer; (iv) allowing the mixture to stand at room temperature for 10 min, then overnight at −20°C; (v) centrifuging at 4°C, 14,000 rpm for 20 min the following day; (vi) carefully transferring 150 μL of the supernatant into a bottle for mass spectrometry analysis; and (vii) preparing quality control (QC) samples by mixing 10 μL from each sample for instrument stability assessment. Samples were stored at −20°C before analysis.

#### 2.5.2 Identification process of lipidomics

Before lipid extraction, a Lipidyzer™ Internal Standard Mix containing 10 major lipid classes and 13 lipid subclasses was added to each sample (AB SCIEX, 5040156). Lipid samples were transferred to the AB SCIEX QTRAP 4500 LC–MS/MS system for analysis. Samples were analyzed on the SCIEX Lipidyzer™ platform, and the multiple response monitoring (MRM) technique was used to target and quantitatively measure 1,100 lipids in 13 subclasses (28, 29). MS data was collected by Analyst (version 1.7, AB SCIEX, MA, United States), and pre-processed by SCIEX OS (version 1.4, AB SCIEX, CA, United States), including peak query, standard curve viewing and so on. The content of lipid metabolites was quantified by the internal standard using the following formula: IS concentration* (sample area/IS area).

#### 2.5.3 Chromatography and mass spectrometry

HPLC-QqQ-MS analytical chromatography was performed under the following conditions: Waters Acquity UPLC BEH HILIC column (100 mm × 2.1 mm; 1.7 μm; Waters, Milford, MA, United States) was used as the stationary phase at 35°C. The mobile phase A contained 10 mmol/L ammonium acetate in 95% acetonitrile solution, and the mobile phase B contained 10 mmol/L ammonium acetate in 50% acetonitrile. The mobile phase gradient consisted of an increase in the concentration of mobile phase B from 0.1 to 20% within 10 min, followed by an increase in mobile phase B to 98% between 10 min and 11 min, which was maintained at 98% for 2 min, and then decreased to 0.1% at 13.1 min. The analysis was terminated at 16 min, and the flow rate was 0.5 mL/min.

Mass spectrometry was conducted with a positive ion mode injection volume of 1 μL and a negative ion mode injection volume of 10 μL. The air curtain gas pressure was set to 35 psi, the atomizer pressure was set to 50 psi, the auxiliary gas pressure was set to 60 psi, and the heating temperature was 500°C. The positive and negative ion mode ion spray voltages were −5,500 V and 5,500 V, the positive and negative ion mode optimized declustering voltages were 80 V and −80 V, the injection voltages were 10 V and −10 V, and the collision chamber injection voltages were 15 V and −15 V.

#### 2.5.4 Methodological verification

Based on the chemical properties and ionization efficiency of lipids, the positive ion mode is more appropriate for the detection of non-polar or moderately polar lipids, such as triacylglycerols (TAGs). In contrast, the negative ion mode is better suited for the detection of polar lipids, such as free fatty acids (FFAs) and glycerophospholipids (30, 31). Therefore, in this study, the samples were scanned twice in positive and negative ions to improve the sensitivity and accuracy of the detection. Instrument reproducibility was assessed by evaluating retention time, peak count, and preliminary detection of major lipid classes, based on the total ion current (TIC) chromatogram of the QC sample. System stability was confirmed with a QC sample relative standard deviation (RSD) of <30% and a feature yield of >80%.

#### 2.5.5 Multivariate data analysis

Pre-processed data were exported, and variables with excessive missing values (based on the 80% rule) were excluded. Multivariate statistical analysis was performed using SIMCA-P 14.1 (Umetrics AB, Umea, Sweden). Firstly, principal component analysis (PCA) was performed. Secondly, orthogonal partial least square discriminant analysis (OPLS-DA) was used to establish the model separately (CON vs. PO, CON vs. PH, PO vs. PH). Then, permutation testing (*n* = 200) was used to determine whether the OPLS-DA model was overfitted. After the OPLS-DA model is successfully verified, an S-plot plot is constructed on the basis of the OPLS-DA model. The lipid molecules were arranged according to the Variable Importance in Projection (VIP) value, and the metabolites with VIP > 1.5 were selected as significant differences. Subsequently, Student’s *t*-test was performed on the selected variables, that is, metabolites having VIP > 1.5 and *p* < 0.05 were considered as potential biomarkers. Additionally, the fold change (FC) and *p*[1] score (derived from the s-plot graph) were utilized to evaluate the variations in compound levels.

### 2.6 Statistical analysis

Statistical analysis was performed using SPSS (version 19.0; SPSS). Data are presented as mean ± SD. One-way ANOVA was used for multiple-group comparisons, and LSD *t*-tests were used for pairwise comparisons. *p* < 0.05 was considered statistically significant.

## 3 Results

### 3.1 Changes in the biochemical indexes of rats

The SUA levels in the PO group were not significantly different from those in the CON group (*p* > 0.05). However, from the 4th week onward, SUA levels in the PH group were significantly higher than those in both the PO and CON groups (*p* < 0.05). The TAG levels in the PO group gradually increased, becoming significantly higher than those in both the PO and CON groups starting from the 4th week (*p* < 0.05). In the PH group, TAG levels showed no significant difference early on, but were significantly higher than in the CON group from the 8th week onwards (*p* < 0.05) (Table 1).

**Table 1:** The changes of biochemical indexes.

	Group	Week 4	Week 8	Week 12
SUA	CON	113.17 ± 8.18	97.83 ± 13.11	101.00 ± 8.27
	PO	105.00 ± 9.94	103.33 ± 10.75	111.17 ± 9.37
	PH	320.67 ± 32.62^ab^	276.00 ± 33.83^ab^	263.33 ± 24.94^ab^
TAG	CON	0.49 ± 0.13	0.57 ± 0.09	0.66 ± 0.19
	PO	0.95 ± 0.23^a^	1.36 ± 0.14^a^	1.77 ± 0.2^a^
	PH	0.72 ± 0.26^b^	1.06 ± 0.16^ab^	0.77 ± 0.12^b^

^aCompared with CON group, P < 0.05; bcompared with PO group, p < 0.05.^

### 3.2 Pathological changes

**Figure 1:**
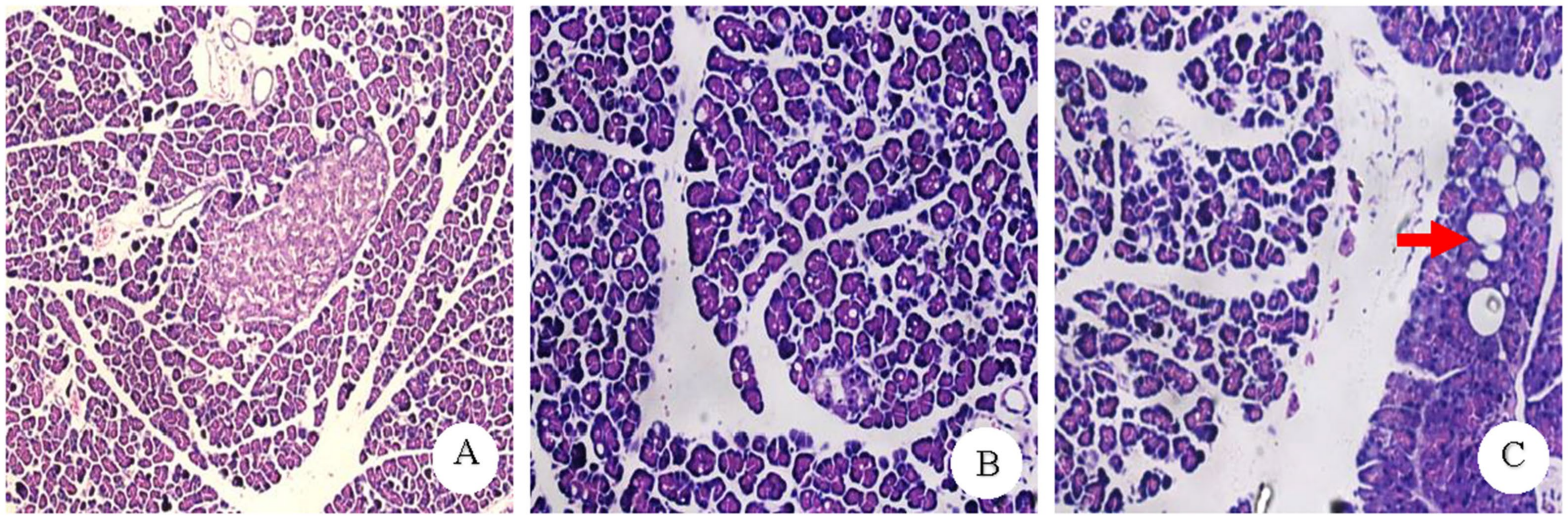
Pathology result plot. **(A)** CON group normal pancreatic pathology (×100). **(B)** PO group pancreatic acinar cells showed diffuse and moderate alveolar vacuole-like changes (×200). **(C)** PH group: some of the fusion into larger vacuoles Kind of change (Shown by the red arrow) (×200).

The morphology and structure of the pancreatic islets and acinar cells in most rats in the CON group were normal, with a pathological score of 4.33 ± 0.16. In the PO group, the pancreatic islet and acinar cells of most rats exhibited minor to moderate fatty changes, with a small amount of adipocyte infiltration in the interstitium and a pathological score of 8.00 ± 0.91. In the PH group, the pancreatic islet and acinar cells of most rats exhibited moderate steatosis in a medium volume, with some fusion generating more marked fatty steatosis, some pancreatic islet cells atrophy, and a small amount of adipocyte infiltration in the interstitium. The pathology score was 10.33 ± 0.69 (Figure 1).

In addition, the pathological scores of the three groups were compared in pairs, and the differences were statistically significant (CON vs. PO, CON vs. PO, PO vs. PH, *p* < 0.05).

### 3.3 Mass spectrometry results

#### 3.3.1 Methodological verification

**Figure 2:**
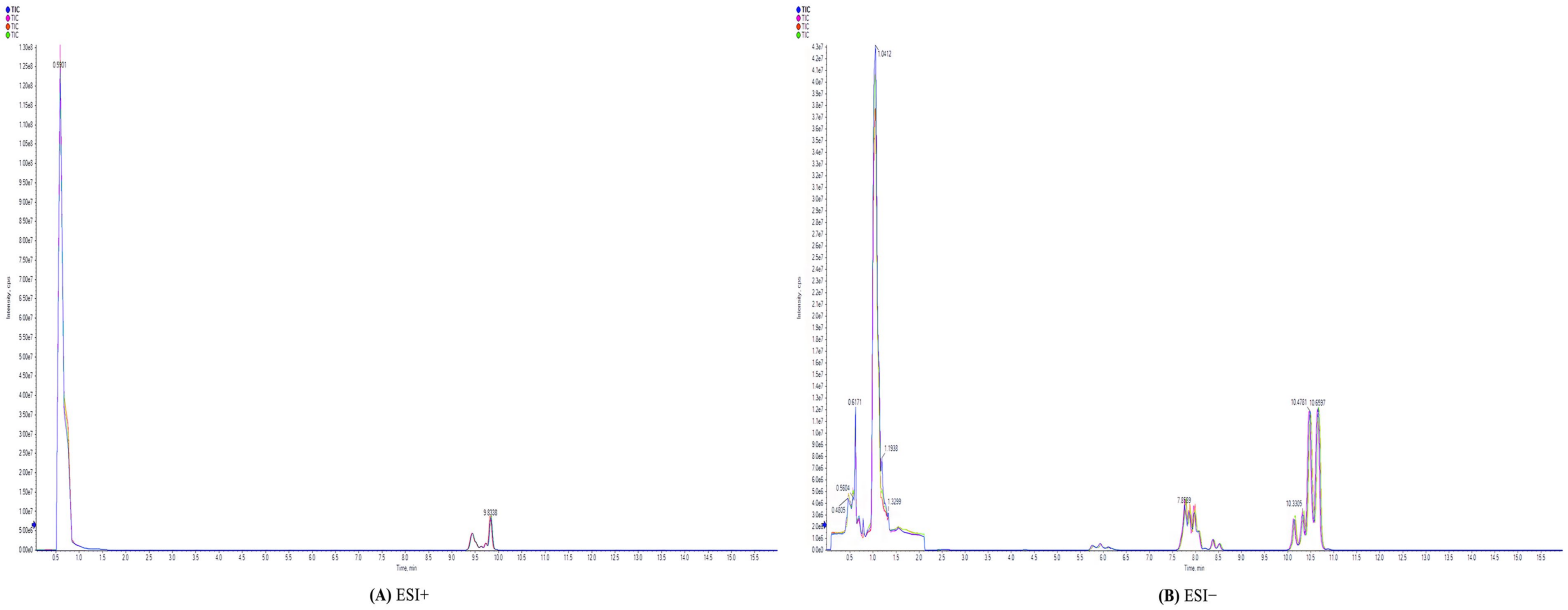
Total ion chromatography (TIC) of QC samples. **(A)** ESI+: positive ion mode. **(B)** ESI−: negative ion mode.

Samples were analyzed by high-resolution mass spectrometry in both positive and negative ion modes. The RSD of QC samples was <30%, ensuring data quality control, and feature yield was >80%. The original total ion flow (TIC) indicates a high concentration in the TIC diagram of the QC samples. The system stability and reliability of the data were confirmed (Figure 2).

#### 3.3.2 Identification of lipid molecules

A total of 884 distinct lipid molecules were identified in both ionization modes. In the positive ion mode, a total of 430 lipids were identified, including 392 TAGs, 12 sphingolipids (SMs), 16 cholesterol esters (CEs), and 10 ceramides (CERs). In the negative ion mode, a total of 454 lipids were identified, including 69 phosphatidylcholines (PCs), 16 lysophosphatidylcholines (LPCs), 105 phosphatidylethanolamines (PEs), 14 lysophosphatidylethanolamines (LPEs), 69 phosphatidylinositol (PIs), 13 lysophosphatidylinositol (LPIs), 66 phosphatidylserines (PS), 16 lysophosphatidylserine (LPSs), 59 phosphatidylglycerols (PGs), 10 lysophosphatidylglycerols (LPGs), and 17 FFAs.

#### 3.3.3 PCA score plots in positive and negative ion mode

**Figure 3:**
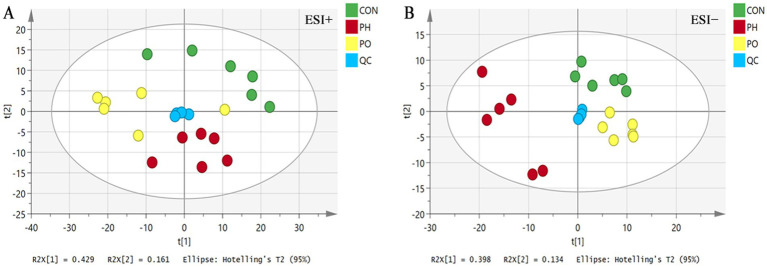
PCA score plots. **(A)** ESI+: positive ion mode. **(B)** ESI−: negative ion mode.

Data were initially analyzed using unsupervised PCA, which can detect the natural grouping of samples without adding any grouping information. PCA analysis showed that QC samples were clustered, indicating that the instrument ran stably during the whole testing process. At the same time, in the positive and negative ion mode, the metabolic profiles of the three groups of samples were significantly different, with *R*^2^*X* = 0.704 in the positive ion mode and *R*^2^*X* = 0.614 in the negative ion mode, suggesting that the PCA model could better explain the metabolic differences among the samples (Figure 3).

#### 3.3.4 Pair-wise comparison of OPLS-DA score plots, permutation testing plots and S-plots

**Figure 4:**
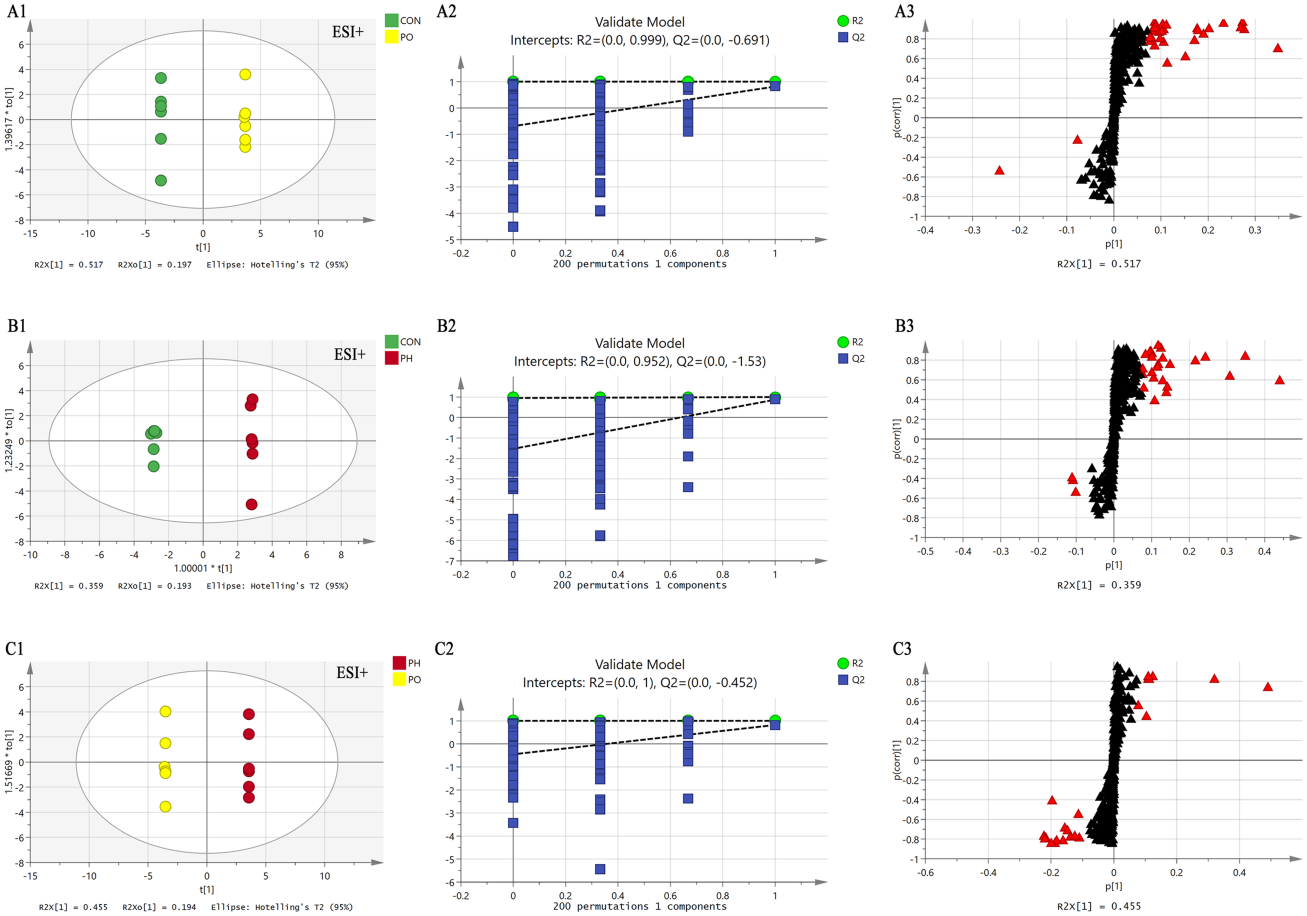
Comparison of OPLS-DA score plots, permutation testing plots and S-plots in positive ion mode. OPLS-DA score plots **(A1,B1,C1)**; S-plots **(A2,B2,C2)**; permutation test plots **(A3,B3,C3)**. As shown in the figure, the models in each group are well fitted, and no overfitting occurs. The lipid molecules labeled with red triangles in the S-plot were selected as potential biomarkers.

**Figure 5:**
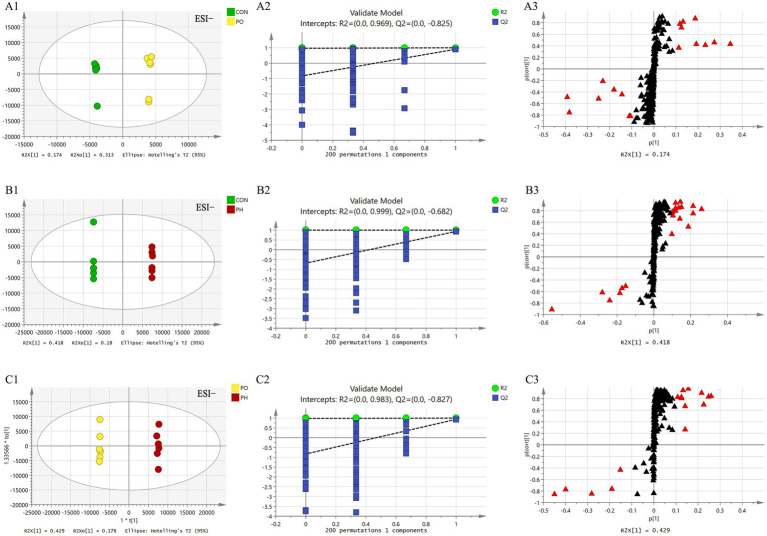
Comparison of OPLS-DA score plots, permutation testing plots and S-plots in negative ion mode. OPLS-DA score plots **(A1,B1,C1)**; S-plots **(A2,B2,C2)**; permutation test plots **(A3,B3,C3)**. As shown in the figure, the models in each group are well fitted, and no overfitting occurs. The lipid molecules labeled with red triangles in the S-plot were selected as potential biomarkers.

Next, orthogonal partial least squares discriminant analysis (OPLS-DA) was employed to construct the model based on group comparisons. The *R*^2^*Y* value was used to evaluate the model’s fit, and *Q*^2^ assessed its predictive ability. A model was considered well-constructed when *R*^2^*Y* was close to 1 and *Q*^2^ > 0.5. To ensure the model was not overfitted, a permutation test (*n* = 200) was performed. When all blue *Q*^2^ values on the left were lower than the initial point on the right, and the *Q*^2^ regression line intersected the vertical axis at or below zero (*Q*^2^ < 0), it confirmed that the model was well-fitted. As shown in the figure, all models were well-fitted and not overfitted. An S-plot was generated based on the OPLS-DA model (CON vs. PO, CON vs. PH, PO vs. PH), and metabolites with VIP > 1.5 and Student’s *t*-test *p* < 0.05 were considered potential biomarkers (Table 2 and Figures 4, 5).

**Table 2:** Evaluation parameters of OPLS-DA models.

Group	ESI+			ESI−		
	*R* ^2^ *X*	*R* ^2^ *Y*	*Q* ^2^	*R* ^2^ *X*	*R* ^2^ *Y*	*Q* ^2^
CON vs. PO	0.98	1.0	0.813	0.933	0.998	0.903
CON vs. PH	0.937	0.999	0.871	0.988	1.0	0.935
PH vs. PO	0.994	1.0	0.819	0.971	1.0	0.938

ESI+: positive ion mode; ESI−: negative ion mode.

#### 3.3.5 Identification of differential metabolites

In the CON and PO groups, 27 potential biomarkers were detected in the positive ion mode, consisting of 26 TAGs and 1 CE, and 7 in the negative ion mode, including 2 PCs, 1 LPC, 1 PE, 2 LPEs, and 1 FFA (Table 3). In the CON and PH groups, 22 biomarkers were identified in the positive ion mode, comprising 17 TAGs, 1 SM, and 4 CEs, and 16 in the negative ion mode, including 8 PCs, 1 PE, 1 LPE, and 6 FFAs (Table 4). In the PH and PO groups, 18 biomarkers were observed in the positive ion mode, consisting of 12 TAGs, 3 SMs, and 3 CEs, and 14 in the negative ion mode, including 8 PCs and 6 FFAs (Table 5). Meanwhile, the conditions for metabolites were as follows: FC > 1, *p*[1] > 0, with an upward trend; conversely, FC < 1, *p*[1] < 0, with a downward trend.

**Table 3:** The list of differential metabolites in CON group and PO group.

NO.	Ion mode	m/z	RT	Compound	*p*-value	*p*[1] score	FC	Trend
1	ESI+	668.6	0.55	CE(18:1)	0.0123	0.3481	5.17	↑
2	ESI+	874.8	0.60	TAG(52:3/FA16:0)	0.0001	0.2761	2.66	↑
3	ESI+	874.8	0.60	TAG(52:3/FA18:2)	0.0001	0.2686	2.91	↑
4	ESI+	876.8	0.60	TAG(52:2/FA16:0)	0.0000	0.2726	5.46	↑
5	ESI+	876.8	0.60	TAG(52:2/FA18:1)	0.0000	0.2333	5.71	↑
6	ESI+	874.8	0.60	TAG(52:3/FA18:1)	0.0001	0.2014	3.14	↑
7	ESI+	900.8	0.60	TAG(54:4/FA18:2)	0.0006	0.1910	3.98	↑
8	ESI+	898.8	0.60	TAG(54:5/FA18:2)	0.0031	0.1708	2.59	↑
9	ESI+	872.8	0.60	TAG(52:4/FA18:2)	0.0333	0.1512	1.56	↑
10	ESI+	900.8	0.60	TAG(54:4/FA18:1)	0.0001	0.1796	4.67	↑
11	ESI+	902.8	0.60	TAG(54:3/FA18:1)	0.0001	0.1770	7.57	↑
12	ESI+	898.8	0.60	TAG(54:5/FA18:1)	0.0038	0.1055	2.59	↑
13	ESI+	876.8	0.60	TAG(52:2/FA18:2)	0.0000	0.1104	3.46	↑
14	ESI+	850.8	0.60	TAG(50:1/FA16:0)	0.0001	0.1061	3.50	↑
15	ESI+	902.8	0.60	TAG(54:3/FA18:2)	0.0002	0.1025	3.99	↑
16	ESI+	898.8	0.60	TAG(54:5/FA20:4)	0.0020	0.0975	2.22	↑
17	ESI+	878.8	0.60	TAG(52:1/FA16:0)	0.0000	0.1042	5.43	↑
18	ESI+	902.8	0.60	TAG(54:3/FA18:0)	0.0003	0.0958	3.80	↑
19	ESI+	848.8	0.60	TAG(50:2/FA16:0)	0.0077	0.0864	2.02	↑
20	ESI+	904.8	0.60	TAG(54:2/FA18:1)	0.0001	0.0870	8.06	↑
21	ESI+	904.8	0.60	TAG(54:2/FA18:0)	0.0000	0.0881	6.01	↑
22	ESI+	924.8	0.60	TAG(56:6/FA20:4)	0.0019	0.0798	2.65	↑
23	ESI+	878.8	0.60	TAG(52:1/FA18:0)	0.0001	0.0839	5.72	↑
24	ESI+	878.8	0.60	TAG(52:1/FA18:1)	0.0000	0.0867	6.65	↑
25	ESI+	898.8	0.60	TAG(54:5/FA16:0)	0.0035	0.0764	2.23	↑
26	ESI+	876.8	0.60	TAG(52:2/FA18:0)	0.0002	0.0808	3.27	↑
27	ESI+	922.8	0.60	TAG(56:7/FA22:6)	0.0016	0.0763	1.82	↑
1	ESI−	480.3	8.40	LPE(18:0)	0.0002	0.1880	1.88	↑
2	ESI−	766.5	6.00	PE(18:0/20:4)	0.0012	0.1308	2.55	↑
3	ESI−	818.6	8.00	PC(16:0/18:1)	0.0093	0.1248	1.57	↑
4	ESI−	452.3	8.50	LPE(16:0)	0.0024	0.1179	1.52	↑
5	ESI−	225.1	0.60	FFA(14:1)	0.0056	−0.3829	0.69	↓
6	ESI−	608.4	10.4	LPC(20:1)	0.0013	−0.1100	0.53	↓
7	ESI−	866.6	7.80	PC(18:1/20:4)	0.0010	−0.1057	0.61	↓

ESI+: positive ion mode; ESI−: negative ion mode. The parameter represents the comparison between the two groups (PO vs. CON).

FC: fold change. *p*[1] score: derived from the s-plot graph. *p*-value: derived from the Student’s *t*-test. (↑): represents rising; (↓): represents falling. TAG, triacylglycerol; SM, sphingolipids; CE, cholesterol esters; PC, phosphatidylcholine; LPC, lysophosphatidylcholines; PE, phosphatidylethanolamine; LPE, lysophosphatidylethanolamine; FFA, free fatty acid.

**Table 4:** The list of differential metabolites in CON group and PH group.

No.	Ion mode	m/z	RT	Compound	*p*-value	*p*[1] score	FC	Trend
1	ESI+	690.6	0.55	CE(20:4)	0.0470	0.4406	1.51	↑
2	ESI+	668.6	0.55	CE(18:1)	0.0007	0.3474	3.09	↑
3	ESI+	666.6	0.55	CE(18:2)	0.0261	0.3084	1.63	↑
4	ESI+	876.8	0.60	TAG(52:2/FA16:0)	0.0011	0.2433	3.52	↑
5	ESI+	876.8	0.60	TAG(52:2/FA18:1)	0.0023	0.2170	3.96	↑
6	ESI+	922.8	0.60	TAG(56:7/FA22:6)	0.0049	0.1484	3.01	↑
7	ESI+	898.8	0.60	TAG(54:5/FA20:4)	0.0012	0.1300	2.29	↑
8	ESI+	874.8	0.60	TAG(52:3/FA18:1)	0.0450	0.1290	1.80	↑
9	ESI+	878.8	0.60	TAG(52:1/FA16:0)	0.0000	0.1247	4.94	↑
10	ESI+	878.8	0.60	TAG(52:1/FA18:0)	0.0000	0.1180	6.33	↑
11	ESI+	850.8	0.60	TAG(50:1/FA16:0)	0.0046	0.1169	3.18	↑
12	ESI+	902.8	0.60	TAG(54:3/FA18:1)	0.0079	0.1160	3.11	↑
13	ESI+	703.6	9.85	SM(16:0)	0.0343	0.1066	1.51	↑
14	ESI+	924.8	0.60	TAG(56:6/FA22:5)	0.0010	0.1002	4.54	↑
15	ESI+	714.6	0.65	CE(22:6)	0.0151	0.0999	1.73	↑
16	ESI+	876.8	0.60	TAG(52:2/FA18:2)	0.0003	0.0998	2.32	↑
17	ESI+	878.8	0.60	TAG(52:1/FA18:1)	0.0002	0.0982	5.78	↑
18	ESI+	876.8	0.60	TAG(52:2/FA18:0)	0.0004	0.0962	3.02	↑
19	ESI+	904.8	0.60	TAG(54:2/FA18:0)	0.0001	0.0955	4.81	↑
20	ESI+	904.8	0.60	TAG(54:2/FA18:1)	0.0004	0.0846	5.28	↑
21	ESI+	898.8	0.60	TAG(54:5/FA16:0)	0.0108	0.0776	1.84	↑
22	ESI+	902.8	0.60	TAG(54:3/FA18:0)	0.0223	0.0770	2.46	↑
1	ESI−	868.6	7.80	PC(18:0/20:4)	0.0008	0.2599	1.90	↑
2	ESI−	840.6	7.91	PC(16:0/20:4)	0.0050	0.2142	1.76	↑
3	ESI−	844.6	8.02	PC(18:0/18:2)	0.0002	0.2119	1.82	↑
4	ESI−	818.6	8.00	PC(16:0/18:1)	0.0003	0.1507	3.28	↑
5	ESI−	846.6	8.02	PC(18:0/18:1)	0.0000	0.1431	3.22	↑
6	ESI−	307.2	0.55	FFA(20:2)	0.0196	0.1397	6.73	↑
7	ESI−	816.6	8.00	PC(16:0/18:2)	0.0004	0.1364	1.62	↑
8	ESI−	766.5	6.00	PE(18:0/20:4)	0.0000	0.1193	4.75	↑
9	ESI−	480.3	8.40	LPE(18:0)	0.0006	0.1175	2.18	↑
10	ESI−	892.6	7.75	PC(18:0/22:6)	0.0020	0.1173	2.18	↑
11	ESI−	277.2	1.25	FFA(18:3)	0.0090	0.1043	2.58	↑
12	ESI−	864.6	7.80	PC(16:0/22:6)	0.0028	0.0996	1.80	↑
13	ESI−	279.2	1.10	FFA(18:2)	0.0000	−0.5544	0.47	↓
14	ESI−	281.2	1.10	FFA(18:1)	0.0311	−0.2799	0.69	↓
15	ESI−	303.2	1.00	FFA(20:4)	0.0048	−0.2398	0.53	↓
16	ESI−	327.2	1.00	FFA(22:6)	0.0304	−0.1837	0.59	↓

ESI+: positive ion mode; ESI−: negative ion mode. The parameter represents the comparison between the two groups (PH vs. CON).

FC, fold change. *p*[1] score: derived from the s-plot graph. *p*-value: derived from the Student’s *t*-test. (↑): represents rising; (↓): represents falling. TAG, triacylglycerol; SM, sphingolipids; CE, cholesterol esters; PC, phosphatidylcholine; LPC, lysophosphatidylcholines; PE, phosphatidylethanolamine; LPE, lysophosphatidylethanolamine; FFA, free fatty acid.

**Table 5:** The list of differential metabolites in PH group and PO group.

No.	Ion mode	m/z	RT	Compound	*p*-value	*p*[1] score	FC	Trend
1	ESI+	690.6	0.55	CE(20:4)	0.0069	0.4906	2.06	↑
2	ESI+	666.6	0.55	CE(18:2)	0.0012	0.3191	1.98	↑
3	ESI+	703.6	9.85	SM(16:0)	0.0005	0.1242	2.08	↑
4	ESI+	813.7	9.45	SM(24:1)	0.0012	0.1116	2.09	↑
5	ESI+	815.7	9.45	SM(24:0)	0.0013	0.1106	2.20	↑
6	ESI+	714.6	0.65	CE(22:6)	0.0006	0.1092	2.61	↑
7	ESI+	874.8	0.60	TAG(52:3/FA16:0)	0.0034	−0.2216	0.56	↓
8	ESI+	872.8	0.60	TAG(52:4/FA18:2)	0.0016	−0.2186	0.46	↓
9	ESI+	874.8	0.60	TAG(52:3/FA18:2)	0.0023	−0.2173	0.54	↓
10	ESI+	898.8	0.60	TAG(54:5/FA18:2)	0.0005	−0.1996	0.28	↓
11	ESI+	872.8	0.60	TAG(52:4/FA16:0)	0.0005	−0.1891	0.47	↓
12	ESI+	900.8	0.60	TAG(54:4/FA18:2)	0.0011	−0.1835	0.33	↓
13	ESI+	900.8	0.60	TAG(54:4/FA18:1)	0.0011	−0.1613	0.36	↓
14	ESI+	876.8	0.60	TAG(52:2/FA16:0)	0.0128	−0.1572	0.64	↓
15	ESI+	874.8	0.60	TAG(52:3/FA18:1)	0.0089	−0.1466	0.57	↓
16	ESI+	902.8	0.60	TAG(54:3/FA18:1)	0.0027	−0.1404	0.41	↓
17	ESI+	896.8	0.60	TAG(54:6/FA18:2)	0.0032	−0.1258	0.39	↓
18	ESI+	898.8	0.60	TAG(54:5/FA18:1)	0.0020	−0.1098	0.40	↓
1	ESI−	868.6	7.80	PC(18:0/20:4)	0.0006	0.2596	1.91	↑
2	ESI−	840.6	7.91	PC(16:0/20:4)	0.0006	0.2475	2.13	↑
3	ESI−	225.1	0.60	FFA(14:1)	0.0116	0.2258	1.58	↑
4	ESI−	844.6	8.02	PC(18:0/18:2)	0.0001	0.2164	1.88	↑
5	ESI−	816.6	8.00	PC(16:0/18:2)	0.0000	0.1553	1.82	↑
6	ESI−	307.2	0.55	FFA(20:2)	0.0171	0.1410	7.90	↑
7	ESI−	846.6	8.02	PC(18:0/18:1)	0.0000	0.1308	2.49	↑
8	ESI−	892.6	7.75	PC(18:0/22:6)	0.0009	0.1256	2.54	↑
9	ESI−	818.6	8.00	PC(16:0/18:1)	0.0014	0.1245	2.09	↑
10	ESI−	864.6	7.80	PC(16:0/22:6)	0.0010	0.1089	2.04	↑
11	ESI−	279.2	1.10	FFA(18:2)	0.0004	−0.4509	0.55	↓
12	ESI−	281.2	1.10	FFA(18:1)	0.0033	−0.3985	0.57	↓
13	ESI−	283.2	1.10	FFA(18:0)	0.0006	−0.2808	0.80	↓
14	ESI−	303.2	1.00	FFA(20:4)	0.0038	−0.1913	0.63	↓

ESI+: positive ion mode; ESI−: negative ion mode. The parameter represents the comparison between the two groups (PH vs. PO).

FC, fold change. *p*[1] score: derived from the s-plot graph. *p*-value: derived from the Student’s *t*-test. (↑): represents rising; (↓): represents falling. TAG, triacylglycerol; SM, sphingolipids; CE, cholesterol esters; PC, phosphatidylcholine; LPC, lysophosphatidylcholines; PE, phosphatidylethanolamine; LPE, lysophosphatidylethanolamine; FFA, free fatty acid.

## 4 Discussion

In this study, high-fat and high-fat and high-uric acid animal models of NAFPD were successfully established, and optimal serum biomarkers were identified using HPLC-QqQ-MS lipidomics. HUA modeling approaches include increasing uric acid production by injecting uric acid or hypoxanthine injections, adenine or yeast feeding, inhibiting uric acid excretion with adenine and ethambutol, blocking uric acid metabolism with potassium oxonate injections (32, 33). Potassium oxonate, a urease inhibitor, is a classic HUA modeling agent, although its stability is limited when used alone, often requiring a combined approach. The yeast-induced HUA model mimics human purine metabolism disorder (34, 35). This study employed a combined yeast and potassium oxonate method. Yeast degradation produces large amounts of purine and pyrimidine, elevating uric acid levels, while potassium oxonate inhibits uric acid breakdown (24). In Sprague–Dawley rats, SUA levels increased and remained stable throughout the experiment. HE staining of pancreatic tissue revealed fatty changes in most islets and acinar cells in both the PO and PH groups, with the PH group showing more severe pathological NAFPD. These results confirm that both high-fat and high-fat-high-uric acid models of NAFPD were successfully established.

Lipidomics is an interdisciplinary field that integrates lipid biology, technology, and medicine (16, 17). Compared with traditional LC–MS, HPLC-QqQ-MS offers superior separation, rapid analysis, and high sensitivity, making it ideal for the rapid identification of lipid compounds. The Lipidyzer™ platform incorporates MRM technology, which enables direct quantitative analysis of lipids by calculating their relative and absolute abundance from mass spectrometry data (28, 29). Lipid compounds are typically classified into eight categories (36): fatty acids, glycerides, glycerophospholipids, sphingolipids (SM), sterol lipids, pregnenolone lipids, glycolipids and polyethylenes. Glycerophospholipids are classified into distinct groups based on their substitution patterns: PC, PE, PI, PS, and PG. This study identified potential serum biomarkers across various groups, as detailed in Tables 3–5. Specifically, 34 potential biomarkers were identified in both the CON and PO groups, 38 in the CON and PH groups, and 32 in the PH and PO groups. These biomarkers primarily consisted of SM, CE, TAG, PC, LPC, PE, LPE and FFA. These findings demonstrate distinct lipid composition differences among the three animal models, with metabolic disorders evident in the PO and PH groups compared with the CON group. These differences may reflect the pathophysiological changes in these rat models.

Triacylglycerol, the most prevalent glycerolipid, showed an upward trend in both the PO and PH groups compared with that in the CON group. Specifically, TAG levels increased by 26% in the PO group and by 17% in the PH group. The PH group showed a 12% decrease relative to the PO group. These results suggest TAG metabolic disturbances in both the PH and PO groups, particularly in the PO group. These findings align with serum analysis, which showed a time-dependent increase in TAG levels, particularly in the PO group. Notably, TAG levels in the PH group were significantly higher than in the CON group after week 4 (*p* < 0.05). Although no significant differences in TAG levels were observed between the PH and CON groups early on, levels in the PH group increased significantly after week 8 (*p* < 0.05). The substantial accumulation of TAG in the pancreas is associated with ectopic lipid deposition, which may contribute to the development of NAFPD in the PO and PH groups (37, 38). HUA can promote lipid deposition (13). TAG may play a crucial role in the pathological progression of NAFPD. Excessive TAG accumulation induces lipid toxicity in pancreatic cells, placing them under continuous stress and causing structural and functional damage of cells through mechanisms such as endoplasmic reticulum stress and oxidative stress. This stress can lead to serious complications such as pancreatitis and pancreatic fibrosis (39, 40). Furthermore, TAG accumulation activates pancreatic inflammation, promoting macrophage infiltration and the release of inflammatory cytokines. This chronic inflammation not only damages pancreatic tissue but may also contribute to pancreatic fibrosis and cancer development (39, 41).

Additionally, studies have reported altered lipase and TAG synthetase activities in the pancreas of patients with NAFPD, resulting in lipid metabolism imbalances. Reduced lipase activity promotes TAG accumulation, while excessive TAG synthetase activation exacerbates this process, creating a vicious cycle that accelerates NAFPD progression (42). Abnormal TAG metabolism is also closely linked to insulin resistance in pancreatic cells. Insulin resistance, characterized by diminished insulin responsiveness and elevated blood glucose, exacerbates NAFPD pathology, creating a feedback loop (40).

Phospholipids are key components of biological cell membranes, with PC, PE, and PI being the most prominent types (43). These lipids can be hydrolyzed by phospholipase A or lecithin-cholesterol acyltransferase to yield LPC, LPE, and fatty acids (44). Additionally, phospholipids, lysophospholipids, and fatty acids can interconvert through the Lands cycle, maintaining lipid metabolism homeostasis. Phospholipase also hydrolyzes arachidonic acid to produce PG, PS, PI, and PE, essential for membrane reconstruction (45). In this study, compared to the CON group, the PO and PH groups showed an overall increase in PC, PE, LPC, and LPE levels. These results suggest that both PO and PH diets induce glycerophospholipid metabolism disorders. Specifically, the PH group exhibited an eightfold increase compared with the PO group, implying that hyperlipidemia and HUA exacerbate glycerophospholipid metabolism dysregulation. This phenomenon may relate to cell membrane damage and reconstruction.

The PC serves as a crucial component of cell membranes. In the context of HUA, PC can be transformed into LPC, LPE, arachidonic acid, and other metabolites related to inflammation through the activity of phospholipase A (46). LPC and LPE are capable of activating the Nuclear Factor-Kappa B (NF-κB) pathway, elevating the levels of Toll-like receptor 4 (TLR4) and phosphorylated Nuclear Factor-Kappa B (p-NF-κB), and resulting in an increased production of inflammatory factors such as Tumor Necrosis Factor-alpha (TNF-*α*) and Interleukin-6 (IL-6), thereby intensifying inflammation and oxidative stress damage (47, 48). Moreover, LPC stimulate mitogen-activated protein kinases (MAPKs), enhance the expression of inflammatory factors, and facilitate the migration and activation of inflammatory cells (49). Macrophages and other immune cells can induce the production of inflammatory cytokines under the influence of LPC, further amplifying the overall inflammatory response (50). Consequently, we hypothesize that the coexistence of hyperlipidemia and hyperuricemia exacerbates NAFPD via enhanced cellular membrane damage and remodeling, potentially mediated by the lipid-induced inflammatory response and increased tissue injury.

The SM plays a crucial role in maintaining biofilm structure and is involved in key signaling processes, including cell growth, differentiation, senescence, and apoptosis (51). In this study, the PH group exhibited a onefold increase in SM compared with the CON group, and a threefold increase compared with the PO group, with no significant differences between the CON and PO groups. These findings suggest that hyperlipidemia-induced lipid metabolism disorders are specifically linked to dysregulated SM metabolism. Previous research has highlighted a close relationship between abnormal sphingomyelin metabolism and fat deposition, as well as inflammation in NAFLD (52). SM are closely associated with inflammatory mediators, and SM metabolites, such as CEs, possess the capacity to induce inflammatory responses while inhibiting superoxide dismutase (SOD) activity. In conditions like neurological diseases and chronic obstructive pulmonary disease, disturbances in sphingosine metabolism may lead to heightened inflammation and oxidative stress, thereby contributing to the progression of these diseases (53, 54).

The HUA is associated with several metabolic disorders, including inflammation, gout, and diabetes (5, 6). Elevated uric acid levels can alter serum lipid composition. Liu et al. (55) first demonstrated that a combination of TAG 18:1–20:0–22:1 and TAG14:0–16:0–16:1 could distinguish asymptomatic patients with HUA from those with gout. Kang et al. (56) found that metabolic disorders of glycerolipids (GLs) and glycerophospholipids were linked to HUA risk. LPC and PC are considered to be important biomarkers for the treatment and prognosis of gouty arthritis (57). Clinical studies have shown that TAGs, SM, and glycerophospholipids (PC, LPC, PG, and LPE) were significantly elevated in plasma lipid profiles in individuals with HUA and gout, particularly among younger patients (58).

High uric acid levels may contribute to lipid metabolism disorders through various mechanisms, including inhibition of Adenosine 5′-monophosphate (AMP)-activated protein kinase (AMPK) signaling and activation of Sterol Regulatory Element Binding Protein 1c (SREBP-1c), which promotes fat synthesis (59, 60). HUA could also induce an inflammatory state by activating the Transforming Growth Factor-*β* (TGF-β) signaling pathway and suppressing the Mammalian Target of Rapamycin (mTOR) signaling pathway, resulting in increased levels of inflammatory factors such as oxidative stress and IL-6 (61). These inflammatory effects can impact lipid metabolism and lead to fat accumulation (62). Additionally, inflammation may disrupt glycerophospholipid metabolism, further exacerbating the inflammatory response and tissue damage. Therefore, it is hypothesized that chronic inflammation, mediated by HUA and lipid metabolites, plays a significant role in the pathophysiology of NAFPD exacerbated by hyperlipidemia combined with HUA.

In summary, NAFPD and HUA are closely linked to lipid metabolism. A 2017 meta-analysis suggested that TAGs could serve as a key serological marker for NAFPD (2). In the present study, the pathological NAFPD score in the PH group was significantly higher than in the PO group. Additionally, our findings implicate glycerophospholipids—PC, LPC, PE, and LPE—as potential serum biomarkers for HUA, which exacerbates NAFPD. These results suggest that glycerophospholipid metabolism plays a crucial role in NAFPD pathophysiology. Furthermore, SM may serve as specific markers indicating the effect of hyperuric acid on NAFPD. The preliminary conclusion of the study suggests that the combination of hyperlipidemia and HUA exacerbates NAFPD, potentially due to disturbances in glycerophospholipid metabolism. This may be attributed to an intensified lipid-mediated inflammatory response, leading to increased tissue injury and further deterioration of cell membrane damage and remodeling. This study offers a novel perspective and theoretical foundation for future extensive clinical and animal experimental investigations. Moreover, it implies the need for heightened focus on lipid metabolism in patients with metabolic disorders like NAFLD, NAFPD and HUA during future clinical practice.

However, there are several limitations to this study. The sample size of animals was relatively small, which limits the ability to fully capture the changes in lipid metabolism profiles. Previous research has indicated that alterations in lipid metabolism during various stages of severe acute pancreatitis may be linked to inflammation and reparative processes (63), while changes in lipid composition as individuals age have been associated with the onset of Alzheimer’s disease (64). Moreover, only serum samples from the final experimental animal were analyzed, failing to account for the dynamic changes in lipid profiles throughout the study. To further validate the relationship between lipid biomarkers and NAFPD, larger sample sizes and large-scale clinical prospective studies are needed.

## 5 Conclusion

In conclusion, hyperlipidemia combined with HUA significantly exacerbates NAFPD. Glycerophospholipids may serve as key biomarkers in this process, potentially linked to a chronic inflammatory response mediated by glycerophospholipids. However, this study has notable limitations. The small sample size restricts the ability to fully capture variations in lipid metabolism profiles. Additionally, the analysis was limited to serum samples from the final experimental animal, preventing the assessment of dynamic lipid profile changes throughout the study. Future research should include larger sample sizes and longitudinal clinical studies to validate the relationship between potential lipid biomarkers and NAFPD.

## Data Availability

Requests to access the datasets should be directed to lslqz@fjmu.edu.cn.
